# Circulating Salicylic Acid and Metabolic Profile after 1-Year Nutritional–Behavioral Intervention in Children with Obesity

**DOI:** 10.3390/nu11051091

**Published:** 2019-05-16

**Authors:** Giulia Vizzari, Maria Chiara Sommariva, Michele Dei Cas, Simona Bertoli, Sara Vizzuso, Giovanni Radaelli, Alberto Battezzati, Rita Paroni, Elvira Verduci

**Affiliations:** 1 Department of Pediatrics, San Paolo Hospital, Department of Health Science, University of Milan, 20142 Milan, Italy; giulia.vizzari@unimi.it (G.V.); mariachiara.sommariva@studenti.unimi.it (M.C.S.); sara.vizzuso@unimi.it (S.V.); giovanni.radaelli@unimi.it (G.R.); 2 Laboratory of Clinical Biochemistry and Mass Spectrometry, San Paolo Hospital, Department of Health Science, Universita’ degli Studi di Milano, 20142 Milano, Italy; michele.deicas@unimi.it (M.D.C.); rita.paroni@unimi.it (R.P.); 3 Dipartimento di Scienze e Tecnologie Alimentari e Microbiologiche (DiSTAM), International Center for the Assessment of Nutritional Status (ICANS), Universita‘ degli Studi di Milano, Via G. Colombo, 60, 20133 Milan, Italy; simona.bertoli@unimi.it (S.B.); alberto.battezzati@unimi.it (A.B.)

**Keywords:** salicylic acid, fruit and vegetables, salicylate intake, childhood obesity

## Abstract

Objectives and Study: Salicylic acid (SA), a phenolic compound produced by plants, may play a beneficial role on health. A pilot study showed that children with obesity had lower serum SA than normal-weight children. The aim of this trial was to evaluate the effect of a 1-year nutritional–behavioral intervention on serum SA levels and to study a possible association between SA levels and metabolic profile changes in children with obesity. Methods: This was an interventional longitudinal observational uncontrolled cohort study. Forty-nine children with obesity, aged >6 years were evaluated. BMI (body mass index) z-scores were calculated. Fasting blood samples were analyzed for lipids, insulin, and glucose. The most significant metabolic variables were calculated. Serum SA was measured using a gas chromatography–mass spectrometry method. The 1-year intervention was based on the promotion of a balanced and normocaloric diet, in accordance with the national guidelines for treatment of childhood obesity. Additionally, behavioral education, based on the revised CALO-RE (Coventry, Aberdeen, and London-REfined) taxonomy, was performed. Results: At the end of intervention, children showed an increase in serum SA levels (mean (Standard Deviation, SD) 0.06 (0.02) vs. 0.09 (0.05) µmol/L; *p* < 0.001), a reduction of BMI z-score (3.14 (0.79) vs. 3.02 (0.82); *p* < 0.001), TyG index (4.52 (0.20) vs. 4.48 (0.23); *p* < 0.001), AIP (atherogenic index of plasma) (0.36 (0.21) vs. 0.27 (0.25); *p* < 0.001), and triglycerides/HDL (high density lipoprotein) cholesterol (2.57 (1.28) vs. 2.18 (1.22); *p* < 0.001) ratio. No statistically significant change in HOMA-IR (homeostasis model assessment index) was observed (4.20 (3.29) vs. 4.03 (2.28)). An association between the longitudinal variation of serum SA and HOMA-IR was found (correlation coefficient: −0.338, *p* = 0.02). Conclusion: Nutritional–behavioral intervention may improve the circulating SA and the metabolic profile in children with obesity. Serum SA could influence mainly glucose metabolism. Further larger studies are needed to evaluate whether a nutritional intervention based on specific advice regarding the quantity and type of fruit and vegetables (FV) consumption could provide benefits in terms of metabolic syndrome.

## 1. Introduction

Salicylic acid (SA) is a metabolite with a phenolic structure produced by plants as a defence system against pathogens and environmental stress [1,2]. Salicylates are present in appreciable amounts in fruits and vegetables, wines, tea, fruit juices, herbs, and spices. Through a systematic review of the literature [3], a food composition database describing median salicylate values for 27 different types of fruits; 21 vegetables; 28 herbs, spices, and condiments; 2 soups; and 11 beverages has been constructed. Using this database, the total salicylate intake in a Scottish population was estimated to be 4.42 and 3.16 mg/day, for males and females respectively, with fruit and vegetables as major sources (25%). However, SA is better known as the principal metabolite and active component of aspirin, an anti-inflammatory drug.

The use of salicylates was introduced before their mechanism of action was known. The main mechanism is represented by the inhibition of cyclooxygenases, responsible for the biosynthesis of prostaglandins by weak bonds. Otherwise, acetylsalicylic acid (aspirin) inhibits cyclooxygenases through a covalent bond. Salicylic acid plays an important role in human and animal metabolism, and although in humans most of it is of exogenous origin, endogenous biosynthesis has also been demonstrated. The main effects of salicylates are analgesia, antipyretic, and anti-inflammatory activity. Low-dose acetylsalicylic acid in chronic administration has an anti-platelet aggregation effect. A study has shown that chronic intake of low-dose aspirin is effective for the prevention of cardiovascular disease and colorectal cancer [1], although in a recent meta-analysis, no association between aspirin consumption and cancer has been reported [4].

Recent studies have also shown that high concentrations of salicylate activate the AMPK (adenosine mono-phosphate kinase) protein (AMP-activated protein kinase), which plays a key role in the regulation of metabolism and cell growth [5].

In the last few years, there has been a growing interest in the role of salicylic acid as a bioactive dietary component with health consequences. It is possible that the beneficial effects of regular fruit and vegetables consumption in healthy subjects could also depend on low chronic SA exposure. Indeed, vegetarians have showed higher circulating SA than the non-vegetarians, and levels similar to those of subjects taking low-dose aspirin (75 mg/day) [6].

To date, only a pilot study on this issue was conducted for paediatric ages. In particular, an association between serum SA and fruit and vegetable consumption in children with obesity and normal-weight children has been studied showing that children with obesity had lower serum SA than normal-weight children [7].

A childhood obesity epidemic has increased rapidly worldwide and is nowadays one of the most serious global public health challenges. Obesity is often associated with chronic low-grade systemic inflammation [8] and related comorbidities [9].

The aim of this trial was to evaluate the effect of a 1-year nutritional–behavioral intervention on serum SA levels and to study a possible association between SA levels and metabolic profile changes in children with obesity. 

## 2. Experimental Section

This was an interventional longitudinal, non-randomized, non-controlled study (https://clinicaltrials.gov/). A group of 51 children with obesity (28 boys and 23 girls) was consecutively recruited among those admitted with a diagnosis of obesity by primary care pediatricians to the Department of Pediatrics, San Paolo Hospital, Milan, Italy, between October 2016 and September 2018, according to the following eligibility criteria: age ≥6 years, weight at birth ≥2500 g and <4000 g, gestational age 37–42 weeks, single birth, and having Caucasian parents. Exclusion criteria were any syndromic, organic, and hormonal conditions besides obesity; use of anti-inflammatory drugs, including aspirin, in the last month; any allergy, food intolerance, or adoption of special diets (gluten-free, vegetarian, vegan diet).

A child was defined obese in accordance with the International Obesity Task Force, i.e., if her/his BMI was above the age- and sex-adjusted BMI (body mass index) Cole’s curve passing through the cut-off of 30 kg/m^2^ at age 18 years [10]. The parents of eligible children or their legal guardian received detailed explanation about the aim of the study and signed a consent form. The Hospital Ethics Committee approved the study protocol and gave ethical clearance.

### 2.1. Anthropometry and Blood Pressure

At recruitment, a medical history was collected from parents using a standardized questionnaire during a personal interview, conducted by the same paediatrician that was in charge of the children’s general examination.

The pediatrician took anthropometric measurements and blood pressure of children, both at recruitment and at the end of intervention, assisted by an experienced operator. Body weight and height were measured using a mechanical column scale (seca 711; seca GmbH & KG, Hamburg, Germany) with an integrated measuring rod (seca 220; seca GmbH & KG). BMI was calculated from the ratio of weight to height squared (kg/m^2^). BMI z-scores were calculated and adjusted for age and sex by using Cole’s LMS method [11] and Italian reference data [12].

Waist circumference (WC) was measured using the measuring tape seca 203 (seca GmbH & KG) to the nearest 0.1 cm at the mid-point between the iliac crest and the lower edge of the ribs at the end of a normal expiration. Triceps skinfold thickness was measured on the left side of the body, using the Harpenden Skinfold Caliper (Chasmors Ltd., London, U.K.) halfway between the acromion process and the olecranon process [13]. Waist-to-height-ratio (WHtR) was calculated as the waist circumference (cm) divided by height (cm).

For the determination of the FM (fat mass) and of the FFM (free fat mass), a Tanita scale for body impedence assessment was used. The segmental body composition analyzer Tanita BC 418 MA (Tanita, Japan) is a single-frequency BIA (bioimpedentiometry) device that uses eight polar electrodes. This device uses a single-point load cell weighing system in the scale platform and can provide separate body mass readings for different segments of the body, such as the right arm, left arm, trunk, right leg, and left leg. An algorithm that incorporates impedance, age, and height was used to estimate the %FM and %FFM [14].

Blood pressure was measured and evaluated according to recommendations of the National High Blood Pressure Education Program Working Group [15].

### 2.2. Biochemistry

Biochemical measurements were scheduled to be performed within 3 ± 1 days of recruitment (baseline) and after 12 months of intervention. Fasting blood samples were taken at 8 h ± 30 min a.m. and immediately analyzed at the hospital laboratory of biochemistry for total cholesterol, HDL (high density lipoprotein) cholesterol, LDL (low density lipoprotein) cholesterol, triglycerides, insulin, and glucose on the cobas^®^ 6000 analyzer series, c501 and e601 modules (Roche Diagnostics GmbH, Hoffmann-La Roche ltd, Mannheim, Germany), which have been recognized as providing robust chemistry and immunochemistry [16]. The homeostatic model assessment of insulin resistance (HOMA-IR), the quantitative insulin sensitivity check (QUICK) index, the pancreatic β-cell function evaluated using HOMA-β%, the triglyceride-glucose index (TyG index), and the atherogenic index of plasma were calculated respectively as: the product of fasting glucose (mmol/L) and fasting insulin (U/mL) divided by 22.5 [17]; 1/(log10 fasting plasma insulin (U/mL) + log10 glucose (mg/dL)) [18]; (20 fasting insulin in (U/mL)/(fasting glucose (mmol/L) − 3.5) [17]; ln(fasting triglycerides (mg/dL) fasting glucose (mg/dL)/2) [19,20]; and log10 of the ratio of plasma triglycerides to HDL-cholesterol [21,22].

### 2.3. Serum Salicylic Acid Determination

Fasting blood samples were taken on the day after the dietary record was completed, at 8 h ± 30 min a.m., since it has been observed that circulating SA is significantly related both to daily fruit and vegetables (FV) intake of the entire previous week and of the last day [23]. Measurement of the SA serum concentration was performed using isotope dilution liquid chromatography tandem mass spectrometry (ID-LC-MS-MS), optimizing the method described by Sirok et al. [24]. In brief, the instrument was a Dionex 3000 UltiMate HPLC (Thermo Fisher Scientific, Rodano (MI), Italy) connected to an AB Sciex 3200 QTRAP LC-MS/MS with electrospray ionization (ESI) TurboIonSpray™ source (AB Sciex S.r.l., Milano, Italy). Samples were analyzed in negative multiple reaction monitoring mode (MRM) using SA-d4 as the internal standard and separated in isocratic on an Inertsil ODS3, 150 × 3.0 mm i.d., 3 μm particle size column (GL Sciences, Tokyo, Japan) with a mixture of acetonitrile and 0.1% formic acid (80:20, *v*/*v*). The mobile phase was delivered at 0.3 mL/min, and the autosampler and the column oven were kept at 5 °C and 20 °C, respectively. This procedure provides high sensitivity (Lower Limit of Quantification, LLoQ 18 nL) and is adequate for population studies as only a small serum quantity is required (≈100 µL).

### 2.4. Dietary Intake

The dietary intake of children was assessed after recruitment using a 7-day dietary record. Parents received complete oral and written instructions about how to weigh food and the recording of such data. They were trained by a dietician to weigh each food offered to the child before consumption and the leftovers, and to record these weights each time. Vegetable intake was quantified, excluding potato and legumes. The 24-h dietary recall was also recorded at the end of the interview to standardize the usual serving size and to evaluate the intake of fruit and vegetables of the day before the blood collection. Quantification and analysis of energy intake and nutrient composition were performed with an ad hoc PC software programme (MetadietaVR, 2013; METEDAsrl, via S.Pellico 4, San Benedetto del Tronto, AP, Italy). Individual salicylate intakes, derived from FV, of the last day before the blood sample were estimated by using a dietary database containing the median salicylate content of 27 types of fruits and 21 vegetables [3], and for those items that were missing, a database developed in 1985 by Swain et al. (1985) [25] was used. 

### 2.5. Intervention

The intervention was based on the promotion of a normocaloric diet, for a 1-year period, balanced for the macronutrient distribution, in accordance with the national guidelines for treatment of childhood obesity [26] and the reference intake levels for nutrients and energy, as recommended by the Italian Society of Human Nutrition [27]. Specifically, it was recommended that children follow a normocaloric diet (daily caloric intake by age and sex [27]), consisting of protein (population reference intake: 0.94–0.99 g/kg∙die, according to age and sex), carbohydrates (45%–60% of energy intake (En)), fat (20%–35% En; <10% En from saturated fatty acids, 5–10% En from polyunsaturated fatty acids, ≤15% En from monounsaturated fatty acids), and fiber (8.4 g/1000 kcal) [28].

This education managing also took into account a range of behaviour change techniques from the revised CALO-RE (Coventry, Aberdeen, and London-REfined) taxonomy (items 1, 2, 5, 6, 7, 15, 20, and 25) [28].

Written guidelines were given to the parents, including general nutritional advice, food choice lists, and recommended average servings for principal food categories according to updated Italian Dietary Reference Values [27] and a Mediterranean diet pyramid, adapted for the pediatric age at our Department on the basis of the pyramid developed for adults [29]. General nutritional advice included increasing fruit and vegetables, legume, and fish intakes while decreasing meat consumption, using more whole grain food, avoiding sugar-sweetened beverages, and limiting sweets. An illustrated brochure explaining potential benefits of daily physical activity was also provided. 

### 2.6. Statistical Analysis

Descriptive data are reported as mean and standard deviation (SD), or median and 25th–75th centile. Normality of the distribution of continuous variables was assessed using the Kolmogorov–Smirnov test. Statistical significance of longitudinal variations was tested using the Student’s *t* test for paired data or the Wilcoxon test, as appropriate. All values of *p* < 0.05 were considered to indicate statistical significance (two-tailed test). The association of SA with HOMA-IR was assessed using the Spearman’s or Pearson’s correlation coefficient, as appropriate. The statistical package for social sciences (SPSS) package version 20.0 (SPSS Inc., Chicago, IL, USA) for Windows (Microsoft, Redmond, WA, USA) was used, for the statistical analysis. 

## 3. Results

Forty-nine children completed the intervention (96.1%). At recruitment, the mean (SD) age and BMI z-score were 10.0 (2.4) years and 3.14 (0.79), respectively. At the end of intervention, a reduction of daily energy intake and a macronutrient redistribution toward the recommended range were observed. In particular, fiber intake (g/1000 kcal) at baseline was lower than the Italian Dietary Reference Values, and had increased by the end of intervention (*p* < 0.001) to reach the reference values [24] (Table 1). An increase of FV intake was observed at the end of intervention (*p* < 0.001).

The triglyceride glucose index decreased through the intervention period (mean variation, ∆, −0.04; 95% CI, (−0.09, 0.02)). An increase in HDL cholesterol (5.77; (4.35, 7.19) mg/dL) and a reduction in triglycerides (−6.08; (−16.17, 4.00) mg/dL), triglycerides/HDL cholesterol ratio (−0.38; (−0.59, −0.17)) LDL/HDL cholesterol ratio (−0.25; (−0.43; −0.07)), and atherogenic index of plasma (AIP) (−0.08; (−0.12, −0.04)) was observed (Table 2).

At the end of intervention children showed lower BMI z-score than at recruitment (mean variation, ∆, −0.10; 95% CI, (−0.30, 0.09)), lower waist-to-height ratio (WHtR) (−0.01; (−0.02, −0.00)), an increase of fat free mass (FFM) (5.07; (37241.04, 42826.53) g), and a decrease of fat free mass % (FFM%) (0.09; (0.40, 2.16)). (Table 3).

**Figure 1:**
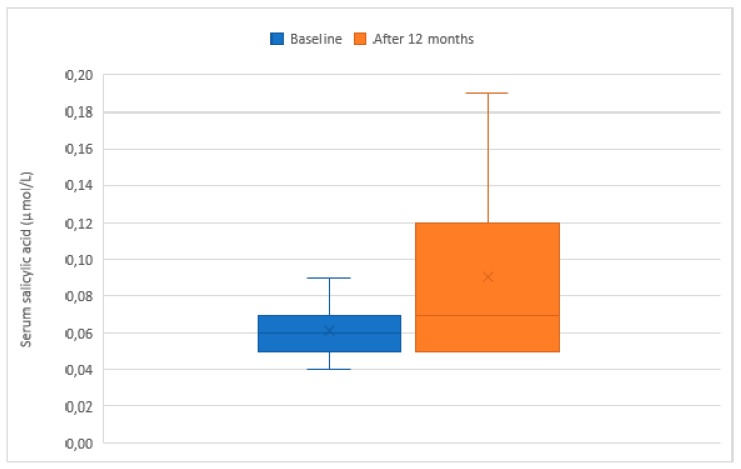
Box-whisker plot of salicylic acid in children with obesity at baseline and at the end of intervention. Significance of difference between groups was *p* < 0.001 (crude) and *p* = 0.072 (adjusted for age, sex, and BMI z-score).

Figure 1 compares serum SA between baseline and the end of the intervention. At the end of intervention, children showed an increase in serum SA levels (mean (SD) 0.06 (0.02) vs. 0.09 (0.05) µmol/L; *p* < 0.001 (crude) and *p* = 0.072 (adjusted for age, sex, and BMI z-score)). Median (25th–75th centile) serum SA was 0.06 µmol/L (0.05–0.07) at baseline and 0.07 µmol/L (0.05–0.12) at the end of intervention. 

Considering the daily introduction of salicylates, no difference was found 1 year after intervention (0.20 (0.16) mg/kg vs. 0.32 (0.17) mg/kg, *p* = 0.273).

No statistically significant change in HOMA-IR was observed (4.20 (3.29) vs. 4.03 (2.28)). An association between longitudinal variation of serum SA and HOMA-IR was found (correlation coefficient: −0.338, *p* = 0.02 and *p* = 0.036 adjusted for sex and Tanner stage).

C-reactive protein (CRP) decreased after 1 year of intervention (4.22 (3.08) vs. 3.91 (2.73) mg/dL; *p* = 0.270).

## 4. Discussion

This is the first study evaluating whether there might be an association between serum SA and metabolic profile in children with obesity after a 1-year nutritional-behavioral intervention.

After a year of intervention, the mean value of salicylic acid increased by around 33% from baseline, but after adjusting for age, sex, and BMI z-score, the increase was not significant.

However, the value found after a year of nutritional intervention was lower than the value observed in normal-weight children (mean value = 0.11 μmol/L) in a previous study [7].

Analyzing the median value of salicylic acid, only the value after nutritional intervention (0.07 μmol/L) was the same as that found in non-vegetarian subjects [6], being however lower than that observed in vegetarian subjects (0.11 μmol/L).

The results of this study underlined that after a 1-year nutritional–behavioral intervention, there was a significant decrease in the BMI z-score of 4%, in accordance with literature [30,31], and a clinically meaningful decrease in waist/height ratio, related to a high risk of developing a metabolic syndrome and cardiovascular diseases [32].

One year after the intervention, the children showed an increase of HDL cholesterol and decrease of triglycerides levels. The AIP index, a predictive parameter of cardiovascular risk that reflects the relationship between protective and atherogenic lipoproteins [21], decreased by the end of the intervention.

The observed effects on the lipid profile are in agreement with the results of previous studies [33,34,35,36]. Furthermore, it should be noted that a systematic review and meta-analysis examining the impact of lifestyle interventions on cardio-metabolic risk factors in overweight and obese children have reported that less than half of the studies showed significant improvements in HDL cholesterol and/or triglyceride levels [37].

Moreover, a reduction of HOMA-IR and TyG indexes was observed after the 1-year lifestyle intervention.

The triglyceride-glucose index is an emergent useful indicator for estimating muscle insulin sensitivity [19,20]. Recent studies examined the usefulness of TyG in pediatric age, suggesting that it could be used in the metabolic evaluation of children/adolescents with obesity [20].

In the present study, an association between the longitudinal variation of serum salicylic acid and the metabolic profile parameters was evaluated.

A negative association between circulating serum salicylic acid and HOMA-IR was found. Rumore et al. [38] showed how treatment with high-dose salicylates may have a key role in glucose metabolism. Indeed, salicylic acid acts by inhibiting NF-kB, a transcription factor that stimulates the inflammatory responses associated with insulin resistance [38] and increasing the expression of PPARGs (peroxisomal proliferator-activated receptor-ɣ) and adiponectin, an adipokine with an insulin-sensitizing effect that is often reduced in subjects with obesity [39].

However, it should be pointed out that the salicylates doses employed in these trials exceed the quantity that can be obtained from diet alone, and that besides the salicylates, other different phenolic compounds with recognized anti-inflammatory and redox-related bioactivity are widely distributed in the plant kingdom [6].

To our knowledge, there are no other human studies aiming toward understanding the effect of nutritional interventions on the longitudinal changes in serum SA. There is only one study performed on rats [40] showing that SA infusion during peppermint cultivation could be used to improve the antidiabetic properties of peppermint infusion per se. The SA-treated peppermint infusions decreased LDL and increased HDL levels, maybe due to its capacity to inhibit pancreatic lipase activity and lipid absorption [40].

As highlighted by the Blacklock study [6], the median levels of salicylic acid in subjects regularly taking low doses of Aspirin (75 mg) were 10.03 μmol/L. This data suggests that circulating levels of serum salicylic acid similar to the observed during low-dose aspirin treatment should result in having anti-inflammatory effects on metabolism. However, at the end of intervention for this present study, the level of SA was 10-fold lower. 

Indeed, it should be noted that, despite the increase in intake of fruits and vegetables at the end of intervention by around of 19%, the average daily consumption of fruit and vegetables of children did not comply with the World Health Organization Guidelines (WHO/FAO 2003), which recommend an intake of 400 g/kg∙die, remaining 37% lower. 

A first limitation of the present study was the minimal effect that the 1-year nutritional–behavioral intervention had on the BMI z-score. Second, the association between HOMA-IR and circulating levels of SA was not so strong. Moreover, another limit is the lack of a control group of children with obesity on a free diet. However, it should be emphasized that the absence of a control group was discouraged by the Hospital Ethics Committee due to the opinion that all children with obesity and their families should have the same opportunity to be educated on dietary recommendations, also taking into account current international guidelines [41]. 

In conclusion nutritional education of families should be promoted, including adequate intake of FV; reducing free sugar and salt intake, starting with avoid sugar-sweetened beverages; and of course, leading a healthy and active lifestyle. Furthermore, the promotion of FV that contains a higher amount of SA, with specific food advice, should be part of the nutritional counselling for children with obesity aiming to increase serum SA to anti-inflammatory levels. 

Further larger studies with appropriate calculation power due to their sample size evaluating the effect of nutritional intervention on the SA longitudinal changes are needed to confirm these results, taking into account both the quantity and the type of FV intake.

## Figures and Tables

**Table 1:** Daily dietary intake of energy, macronutrients, fibre and fruit and vegetables at baseline and at the end of intervention.

Variable	Baseline (*n* = 49)	End of Intervention (*n* = 49)	*p* *	Reference Values ^a^
Average of the 7-day record Energy				
kcal	2324.08 (534.50)	1743.08 (439.47)	<0.001 *	1380–3170 kcal/day depending on age and sex
kJ	9728.61 (2902.43)	7296.53 (2386.26)		
Protein				
g	98.28 (35.96)	70.60 (43.85)	<0.001 *	
% Energy	17 (3)	16 (3)	0.102	<15% Energy
Carbohydrates				
g	321.20 (96.58)	263.87 (62.42)	<0.001 *	
% Energy	54 (6)	56 (5)	0.076	45–60% Energy
*Fat*				
g	81.84 (29.88)	55.44 (26.12)	<0.001 *	
% Energy	32 (5)	28 (5)	<0.001 *	20–35% Energy
*Fiber*				
g	17.34 (5.95)	18.87 (6.74)	0.236	
g/1000 kcal	7.97 (1.26)	10.59 (1.67)	<0.001 *	8.4 g/1000 kcal
Fruit and vegetables				
Average of the 7-day record				
Amount (g)	203.50 (91.17)	252.27 (93.74)	<0.001 *	≥ 400 g/day
Last day record				
Amount (g)	235.68 (96.72)	313.43 (98.03)	<0.001 *	≥ 400 g/day

Values are mean (SD). ^a^ Energy, macronutrients, and fiber (SINU 2014); amount of fruit and vegetables (WHO/FAO 2003). * Statistically significant (*p* < 0.05).

**Table 2:** Glucose metabolism and lipid profile at baseline and after 12 months of intervention. Values are mean (SD) and median (25th–75th centile).

	Baseline (*n* = 49)	End of Intervention (*n* = 49)	
Variable	Mean (SD); Median (25th–75th centile)	Mean (SD); Median (25th–75th centile)	*p* *
Glucose metabolism Glucose (mg/dL)			
	82.64 (7.92); 82.50 (78.00–88.50)	81.98 (8.53); 82.00 (75.00–86.50)	0.978
Insulin (nU/L)	19.93 (13.98); 17.30 (10.70–22.68)	19.69 (11.05); 15.20 (12.05–28.05)	0.525
HOMA-IR	4.20 (3.29); 3.42 (2.06–5.19)	4.03 (2.28); 3.23 (2.37–6.02)	0.460
HOMA-β%	391.55 (232.52); 288.00 (256.80–529.07)	462.75 (441.41); 346.63 (198.81–477.00)	0.069
QUICK index	0.32 (0.03); 0.32 (0.30–0.34)	0.32 (0.03); 0.32 (0.29–0.33)	0.959
TyG index	4.52 (0.20); 4.55 (4.40–4.59)	4.48 (0.23); 4.49 (4.35–4.62)	<0.001 *
Lipid profile Total cholesterol (mg/dL)			
	159.23 (19.17); 161.00 (144.00–176.50)	160.89 (24.03); 157.00 (142.00–181.50)	0.201
LDL cholesterol (mg/dL)	92.19 (18.15); 92.50 (81.00–106.00)	90.94 (30.80); 91.00 (66.00–113.00)	0.930
HDL cholesterol (mg/dL)	45.92 (9.43); 43.00 (38.00–55.00)	51.53 (10.83); 48.00 (43.00–58.00)	<0.001 *
Triglycerides (mg/dL)	111.58 (46.66); 99.00 (82.00–119.50)	104.55 (48.25); 95.00 (71.50–137.00)	<0.001 *
Triglycerides/HDL cholesterol	2.57 (1.28); 2.18 (1.69–3.00)	2.18 (1.22); 2.20 (1.21–2.84)	<0.001 *
LDL/HDL cholesterol	2.13 (0.77); 1.97 (1.49–2.48)	1.89 (0.82); 1.69 (1.15–2.69)	<0.001 *
AIP	0.36 (0.21); 0.34 (0.23–0.48)	0.27 (0.25); 0.34 (0.08–0.45)	<0.001 *

HOMA-IR, homeostasis model assessment of insulin resistance; QUICK, quantitative insulin sensitivity check; TyG, triglyceride glucose; LDL, low-density-lipoprotein; HDL, high-density-lipoprotein; AIP, atherogenic index of plasma. International System of Units conversion factors: to convert glucose, divide values by 0.0555; to convert insulin, divide values by 6.945; to convert cholesterol, divide values by 0.0259; to convert triglycerides, divide values by 0.0113. * Statistically significant (*p* < 0.05).

**Table 3:** Anthropometric parameters at baseline and after 12 months of intervention. Values are mean (SD) and median (25th–75th centile).

	Baseline (*n* = 49)	End of Intervention (*n* = 49)	
Variable	Mean (SD); Median (25th–75th centile)	Mean (SD); Median (25th–75th centile)	*p* *
Anthropometric parameters			
BMI z-score	3.14 (0.79); 2.92 (2.72–3.70)	3.02 (0.82); 2.77 (2.40–3.73)	<0.001 *
Tricipital skinfold	30.79 (5.26); 29.40 (27.00–35.00)	31.84 (5.59); 34.00 (27.00–37.00)	0.293
WHtR	0.62 (0.06); 0.62 (0.57–0.64)	0.61 (0.07); 0.60 (0.56–0.66)	<0.001 *
FM (gr)	23581.82 (11582.97); 20650.00 (16450.00–25600.00)	25546.941 (12670.54); 24050.00 (18250.00–32700.00)	0.253
FFM (gr)	34722.73 (8102.45); 34900.00 (28100.00–43300.00)	39797.73 (7941.49); 42100.00 (32700.00–47225.00)	<0.001 *
FM (%)	38.80 (7.30); 38.00 (33.85–39.80)	38.65 (7.18); 38.10 (33.40–42.05)	0.126
FFM (%)	61.48 (7.49); 63.40 (60.25–66.20)	61.39 (7.17); 61.90 (57.95–66.60)	<0.001 *
PAS (mmHg)	116.85 (11.22); 117.00 (109.75–127.00)	118.25 (10.40); 120.00 (111.00–126.00)	0.939
PAD (mmHg)	61.24 (10.31); 58.00 (56.00–62.00)	64.98 (8.71); 62.50 (58.00–70.00)	0.146

BMI, body mass index; WHtR, waist-to-height ratio; FM, fat mass; FFM, fat free mass; PAS, systolic blood pressure; PAD, diastolic blood pressure. * Statistically significant (p < 0.05).
